# Dietary Chinese herbal formula supplementation improves yolk fatty acid profile in aged laying hens

**DOI:** 10.1080/01652176.2024.2319828

**Published:** 2024-02-25

**Authors:** Zhihua Li, Chengwen Meng, Md. Abul Kalam Azad, Wencao Lin, Jue Gui, Yadong Cui, Wei Lan, Qinghua He, Xiangfeng Kong

**Affiliations:** a Key Laboratory of Agro-ecological Processes in Subtropical Regions, Hunan Provincial Key Laboratory of Animal Nutritional Physiology and Metabolic Process, National Engineering Laboratory for Pollution Control and Waste Utilization in Livestock and Poultry Production, Institute of Subtropical Agriculture, Chinese Academy of Sciences, Changsha, China; b Department of Food Science and Engineering, College of Chemistry and Environmental Engineering, Shenzhen University, Shenzhen, China; c School of Biology and Food Engineering, Fuyang Normal University, Fuyang, China

**Keywords:** Chinese herbal formula, fatty acids, egg yolk, aged laying hens, targeted metabolomics

## Abstract

Chinese herbal formula (CHF) has the potential to improve the performance of aged laying hens through integrated regulation of various physiological functions. The present study aimed to investigate the effects of dietary CHF supplementation on the yolk fatty acid profile in aged laying hens. A total of 144 healthy 307-day-old Xinyang black-feather laying hens were randomly allocated into two groups: a control group (CON, fed a basal diet) and a CHF group (fed a basal diet supplemented with 1% CHF; contained 0.30% *Leonurus japonicus* Houtt., 0.20% *Salvia miltiorrhiza* Bge., 0.25% *Ligustrum lucidum* Ait., and 0.25% *Taraxacum mongolicum* Hand.-Mazz. for 120 days). The fatty acid concentrations in egg yolks were analyzed using a targeted metabolomics technology at days 60 and 120 of the trial. The results showed that dietary CHF supplementation increased (*p* < .05) the concentrations of several saturated fatty acids (SFA, including myristic acid and stearic acid), monounsaturated fatty acids (MUFA, including petroselinic acid, elaidic acid, trans-11-eicosenoic acid, and cis-11-eicosenoic acid), polyunsaturated fatty acids (PUFA, including linolelaidic acid, linoleic acid, γ-linolenic acid, α-linolenic acid, 11c,14c-eicosadienoic acid, eicosatrienoic acid, homo-γ-linolenic acid, arachidonic acid, and docosapentaenoic acid), and fatty acid indexes (total MUFA, n-3 and n-6 PUFA, PUFA/SFA, hypocholesterolemic/hypercholesterolaemic ratio, health promotion index, and desirable fatty acids) in egg yolks. Collectively, these findings suggest that dietary CHF supplementation could improve the nutritional value of fatty acids in egg yolks of aged laying hens, which would be beneficial for the production of healthier eggs to meet consumer demands.

## Introduction

Egg yolks have high nutritional value and are one of the most important sources of essential fatty acids in daily diet, which are of great significance for the growth and development of the human body, protection from diseases, and immunity regulation (Kovacs-Nolan et al. 2005). Different fatty acids induce specific physiological effects on human health. However, due to the great intensification of poultry breeding, several metabolic disorders occur in laying hens after the peak-laying period. Metabolic disorders during the laying period lead to poor digestibility, absorption, and utilization of nutrients, abnormal accumulation of liver and abdominal fat, and the declined reproductive system function, thus inducing a negative influence on the health, performance, and egg quality of laying hens (Liu et al. 2018; Gu et al. 2021; Dai et al. 2022). In addition, the late laying period occupies a high proportion of time in the total reproduction cycle of the laying period of hens. Therefore, maintaining the production performance and improving the quality and nutritional value of egg yolks are important strategies to ameliorate the productivity of aged laying hens.

Recently, the application of Chinese herbs as antibiotic alternatives in poultry production has received special attention. Studies have confirmed that Chinese herbs and their extracts have the potential to improve the organism health, production performance, and product quality in poultry (Obianwuna et al. 2022; Chen et al. 2023), with the advantages of comparatively higher safety and lower side effects (Hou and Jiang 2013). Although most of the previous findings partially proved the effectiveness of a single Chinese herb supplementation in diets (Abbood et al. 2017; Lv et al. 2019; Zhang et al. 2023), considering Chinese herbal formula (CHF) as a whole can regulate various physiological functions in multiple ways and achieve integrated effects to confirm the complexity of actual production conditions according to the guidelines of traditional Chinese medicine theory (Wang et al. 2021). Meanwhile, the application of Chinese herbs as additives was limited due to low utilization rate, poor palatability, and limited expected outcome. The ultrafine pulverization technology helps to fully dissolve the bioactive components in the cells of Chinese herbs and improve the drug efficacy, thus reducing the cost (Liu et al. 2022). However, only a few studies have investigated the effects of dietary ultrafine CHF on egg yolk quality.

Our previous studies indicated that dietary ultrafine CHF supplementation significantly improved the egg production rate and eggshell strength, increased the plasma estradiol level, and enhanced hepatic anti-oxidation ability during the late laying period (Han, Lin, et al. 2023; Han, Lan, et al. 2023); thus, prolonging the ‘service period’ of laying hens. However, the effects of dietary CHF on the nutritional values of eggs remained unknown. Therefore, we hypothesized that CHF supplementation in laying hen’s diet might enhance the nutritional value of eggs. Thus, the present study evaluated the effects of dietary CHF supplementation on egg yolk fatty acid profile in aged laying hens, aiming to provide a theoretical reference for the application of CHF in poultry production.

## Materials and methods

### Preparation of Chinese herbal formula

The Chinese herbs, including *Leonurus japonicus* Houtt. (LJ), *Salvia miltiorrhiza* Bge. (SM), *Ligustrum lucidum* Ait. (LL), and *Taraxacum mongolicum* Hand.-Mazz. (TM), were purchased from Henan Kangxing Pharmaceutical Co., Ltd. (Zhengzhou, China). After washing and drying, LJ, SM, LL, and TM were weighed at a ratio of 3:2:2.5:2.5, pulverized, sieved through a 400-mesh sieve, and sterilized for further use.

### Animals and experimental treatments

A total of 144 healthy 307-day-old Xinyang black-feather laying hens were randomly allocated into two groups, with eight replicates per group and nine hens per replicate. The birds in the control group were fed a basal diet (CON group), and the birds in the experimental group were fed a basal diet supplemented with 1% CHF (CHF group) according to our previous findings (Han et al. 2023), after seven days of acclimation. The composition and nutrient levels of the basal diet were as described by the Xinyang black-feather laying hens management guidelines (Shanghai Poultry Breeding Company, Ltd., Shanghai, China) and presented in Table 1. Total fatty acid composition of control and CHF diets is shown in Table 2. The animal experiment was carried out at the Fuyang Hui Siyuan Ecological Agriculture Co., Ltd., Anhui, China. During the trial, the laying hens were fed twice daily (09:00 and 15:00), and water was available *ad libitum*. The laying hens were subjected to a 16-h light per day and kept in a temperature (18 − 23 °C) and humidity (55%−65%) controlled room. The experiment lasted 120 d.

**Table 1.** Composition and nutrient levels of the basal diet, % air-dried.

Ingredients	Content	Nutrient component^b^	Content
Corn	62.59	Metabolizable energy (MJ/kg)	11.25
Soybean meal	23.88	Crude protein	14.23
Limestone powder	7.94	Ether extract	9.66
Soybean oil	0.49	Calcium	3.51
Methionine	0.10	Phosphorus	0.34
Premix^a^	5.00	Lysine	0.71
Total	100.00	Methionine	0.37

^a^ The premix provided the following per kg of diets: vitamin A 7,000 IU, vitamin D_3_ 2,500 IU, vitamin E 24 mg, vitamin Κ_3_ 9 mg, VB_1_ 3.15 mg, VB_2_ 10 mg, VB_6_ 7 mg, VB_12_ 0.35 mg, nicotinic acid 50 mg, D-pantothenic acid 25 mg, folic acid 2.5 mg, D-biotin 0.25 mg, choline chloride 450 mg, Fe (as ferrous sulfate) 100 mg, Cu (as copper sulfate) 15 mg, Mn (as manganese sulfate) 90 mg, Zn (as zinc sulfate) 100 mg, I (as potassium iodide) 3.5 mg, Se (as sodium selenite) 0.45 mg, and phytase 150 IU.

^b^ Crude protein and ether extract were measured values, while the others were calculated values.

**Table 2.** Total fatty acid composition from diets control (CON) and supplemented with Chinese herbal formula (CHF, 1.0%).

Fatty acids (% of the total fatty acids)	CON group	CHF group
Hexanoic acid, C6:0	0.15	0.18
Lauric acid, C12:0	0.12	0.21
Myristic acid, C14:0	0.34	0.45
Palmitic acid, C16:0	18.59	20.88
Palmitoleic acid, C16:1n-7c	0.19	0.20
Heptadecanoic acid, C17:0	0.16	0.19
Stearic acid, C18:0	5.25	7.33
Oleic acid, C18:1n-9c	25.02	23.63
Linoleic acid, C18:2n-6c	47.17	43.51
α-Linolenic acid, C18:3n-3	1.81	2.21
Arachidic acid, C20:0	0.47	0.48
5Z,8Z,11Z,14Z,17Z-Eicosapentaenoic acid, C20:5n-3	0.23	0.22
cis-11-Eicosenoic acid, C20:1n-9c	0.29	0.30
Docosanoic acid, C22:0	0.21	0.22

The animal study was approved by The Ethics Committee of the Institute of Subtropical Agriculture, Chinese Academy of Sciences (No. ISA-2018-071).

### Sample collection

At days 60 and 120 of the trial, eight laying hens from each group (one hen per replicate) with similar body weight were selected for sampling after 12 h fasting. Blood samples (10 mL) were collected from the wing vein, heparinized, and centrifuged at 3500 × g for 10 min at 4 °C to obtain plasma and then stored at −80 °C until further analysis of biochemical parameters. Meanwhile, eight eggs from each group (one egg per replicate) were randomly selected, broken to collect egg yolk samples, and then immediately transferred at −80 °C until further analysis.

### Determination of plasma biochemical parameters

The plasma concentrations of total protein (TP), albumin (ALB), total cholesterol (TC), and triglyceride (TG) were measured by a Roche automatic biochemical analyzer (Cobas c311; F. Hoffmann-La Roche Ltd., Basel, Switzerland) using the commercially available kits (F. Hoffmann-La Roche Ltd., Basel, Switzerland).

### Determination of total cholesterol in egg yolks

The TC content in egg yolks at days 60 and 120 of the trial was measured using the total cholesterol kit (Nanjing Jiancheng Bioengineering Institute, Nanjing, China) following the manufacturers’ instructions.

### Targeted metabolomics analysis for medium- and long-chain fatty acids profiles in egg yolks

Egg yolk samples (100 mg) were placed in 2 mL centrifuge tubes, homogenized for 1 min with 800 µL 50% acetonitrile water, and centrifuged at 10,000 × g for 15 min at 4 °C to obtain the supernatant. Approximately 400 µL of supernatant was added into 200 µL 3-NPH (200 mM) and 200 µL EDC (120 mM; containing 6% pyridine and 400 ng/µL acetic acid-d3), vortexed for 1 min, incubated at 40 °C for 1 h (shaken once each 5-min), and centrifuged at 10,000 × g for 15 min at 4 °C. The supernatant was filtered through a 0.22-μm membrane and diluted 10-fold with 50% acetonitrile-water (containing internal standard 100 ng/mL) for liquid chromatography-tandem mass spectrometry (LC-MS) analysis.

The LC-MS analysis was performed on Waters Acquity Ultra-high performance LC (Waters Corporation, Milford, MA, USA) and AB SCIEX 5500 QTRAP -MS (AB Sciex Pte., Ltd., Framingham, MA, USA). The Acquity UPLC HSS T3 column (1.80 µm, 2.10 × 100 mm; Waters Corporation, Milford, MA, USA) was used to carry out the chromatographic separation of all the samples, with the column temperature maintained at 40 °C. The chromatographic separation conditions were as follows: mobile phase A, ultra-pure water with 0.01% formic acid; mobile phase B, acetonitrile; flow rate, 0.30 mL/min; and injection volume, 2 μL. The gradient conditions of the metabolic processes were 60%, 20%, 1%, 1%, 60%, and 60% at 1, 5, 16, 17, 18, and 20 min, respectively. The MS parameters were as follows: curtain gas flow rate, 20 Arb; collision gas flow rate, 9 Arb; ion spray voltage, −4200 V; ion source temperature, 450 °C; ion source gas 1 flow rate, 35 Arb; and ion source gas 2 flow rate, 35 Arb. The multiple reaction monitoring (MRM) modes were selected for MS analysis. The data were obtained for further metabolomics and statistical analysis.

Based on the particular concentrations of medium- and long-chain fatty acids (MCFA and LCFA) in egg yolks, the following indexes were calculated: saturated fatty acids (SFA), monounsaturated fatty acids (MUFA), polyunsaturated fatty acids (PUFA), n-3 PUFA, and n-6 PUFA. In addition, the indexes of fatty acids related to health, including atherogenicity index (AI), thrombogenic index (TI), hypocholesterolemic/hypercholesterolemic (HH) ratio, health promotion index (HPI), and desirable fatty acids (DFA) (Medeiros et al. 2014; Omri et al. 2019; Batkowska et al. 2021) were calculated. The calculations were as follows: AI = (4 × C14:0 + C16:0)/(MUFA + PUFA), TI = (C14:0 + C16:0 + C18:0)/(0.5 × MUFA + 0.5 × n-6 PUFA + 3 × n-3 PUFA + n-3/n-6 PUFA), HH ratio = (C18:1n-9 + C18:2n-6 + C20:4n-6 + C18:3n-3 + C20:5n-3 + C22:5n-3)/(C14:0 + C16:0), HPI = (MUFA + PUFA)/(C12:0 + 4 × C14:0 + C16:0), and DFA = C18:0 + MUFA + PUFA.

### Targeted metabolomics data analysis

The methodology of metabolomics data analysis was conducted as previously described with some modifications (Liu et al. 2022; Zhu et al. 2022). Briefly, the raw data were collected from the LC-MS analyzer and pre-processed using the Compound Discoverer software (Thermo-Fisher Scientific, Waltham, MA, USA) to normalize the sum of the peak area before analysis. Then, the standard curves were established to calculate the concentration of each item using the Excel 2022 software according to the interior label. Principal component analysis (PCA) was performed using the SIMCA software (V14.1; Sartorius Stedim Data Analytics AB, Umea, Sweden) to visualize the distribution of the samples. Orthogonal projection to latent structures-discriminant analysis (OPLS-DA) was applied to visualize group separations and identify significant metabolites. The *p* value <.05 was considered as statistical significance. The Kyoto Encyclopedia of Genes and Genomes (KEGG) and MetabolAnalyst databases were applied for pathway enrichment analysis (http://www.genome.jp/kegg/).

### Statistical analysis

All experimental data were analyzed by two-way analysis of variance (ANOVA) using the general linear model procedure of SPSS statistical software (SPSS 22.0; IBM Inc., Chicago, IL, USA). The statistical model includes the effects of diets, time, and their interactions. Differences were considered significant if *p* < .05. Tukey’s multiple comparison tests were used to differentiate significantly different among treatments according to the interaction of the main effects (*p* < .05). Data are presented as means with standard error of the mean (SEM).

## Results

### Effects of dietary CHF supplementation on plasma biochemical parameters of aged laying hens

The effects of dietary CHF supplementation on the plasma biochemical parameters of aged laying hens are presented in Table 3. Dietary CHF supplementation did not affect (*p* > .05) plasma ALB, TC, TG, and TP levels at days 60 and 120 of the trial compared with the CON group. In addition, there were no significant time and diet interactions (*p* > .05) for plasma ALB, TC, TG, and TP levels.

**Table 3.** Effects of dietary supplementation with Chinese herbal formula (CHF) on plasma biochemical parameters of aged laying hens.

Items	Day 60 of the trial		Day 120 of the trial		SEM	*p*-values		
	CON	CHF	CON	CHF		Time	Diet	Time × Diet
ALB (g/L)	23.54	24.31	21.62	23.05	0.573	.183	.351	.779
TC (mmol/L)	3.08	2.98	2.90	2.93	0.182	.762	.930	.856
TG (mmol/L)	11.26	12.14	11.68	12.99	0.436	.484	.230	.810
TP (g/L)	61.56	59.85	57.43	56.20	1.154	.104	.529	.918

Data are presented as means with their SEM (*n* = 8). CON, birds fed a basal diet; CHF, birds fed a basal diet supplemented with 1% Chinese herb formula; ALB: albumin; TC: total cholesterol; TG: triglyceride; TP: total protein.

### Effects of dietary CHF supplementation on total cholesterol level in egg yolks

**Figure 1.**
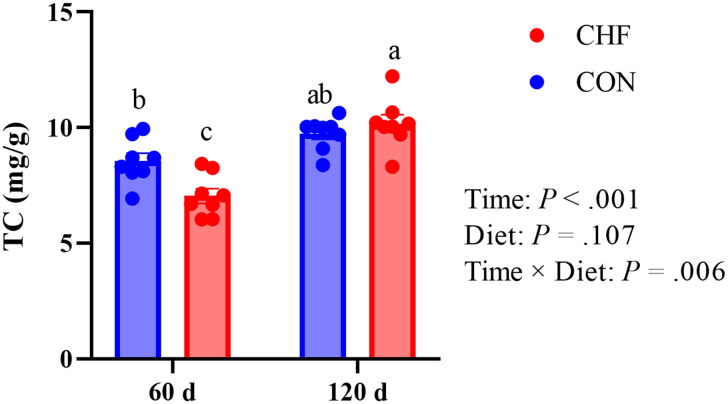
Effects of dietary supplementation with Chinese herbal formula (CHF) on total cholesterol (TC) concentration in the egg yolk of aged laying hens. Data are shown as means with their SEM (*n* = 8). ^a−c^ Different letters indicate significant differences among the four groups (*p* < .05). CON, birds fed a basal diet; CHF, birds fed a basal diet supplemented with 1% Chinese herb formula.

The TC level in egg yolks is shown in Figure 1. Dietary CHF supplementation decreased (*p* < .05) TC level in the egg yolk at day 60 of the trial compared with the CON group. The TC level in the egg yolk of the CHF group was increased (*p* < .05) with age. There was an interaction between time and diet on the TC level in the egg yolk.

### Effects of dietary CHF supplementation on medium- and long-chain fatty acids profiles in egg yolks

**Figure 2.**
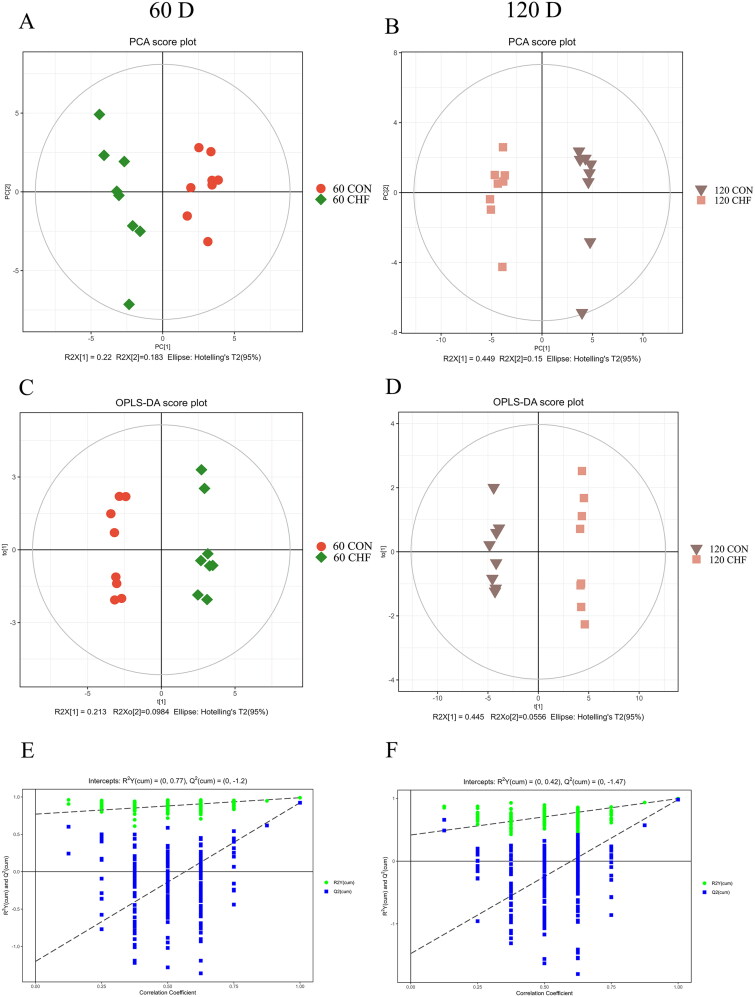
PCA and OPLS-DA models to evaluate fatty acids data in the egg yolk of aged laying hens (*n* = 8). The principal component analysis (PCA; A and B) and orthogonal projections to latent structures-discriminant analysis (OPLS-DA; C and D) score plots of the fatty acids in egg yolks and permutation test of the OPLS-DA model (E and F) for the CHF vs. CON groups at days 60 and 120 of the trial. CON, birds fed a basal diet; CHF, birds fed a basal diet supplemented with 1% Chinese herb formula. CHF: Chinese herbal formula.

After searching the corresponding fragment and peak area and exporting the raw data, the negative ion mass spectrum identified the characteristics of 52 species of targeted MCFA and LCFA. The unsupervised PCA (Figure 2(A,B)) and supervised OPLS-DA (Figure 2(C,D)) showed that the fatty acids distribution of the CHF and CON groups was significantly separated at days 60 and 120 of the trial, indicating that MCFA and LCFA metabolome profiles of the samples were different after dietary CHF supplementation. The results of the permutation test showed that the OPLS-DA model was successfully established (Figure 2(E,F)).

**Figure 3.**
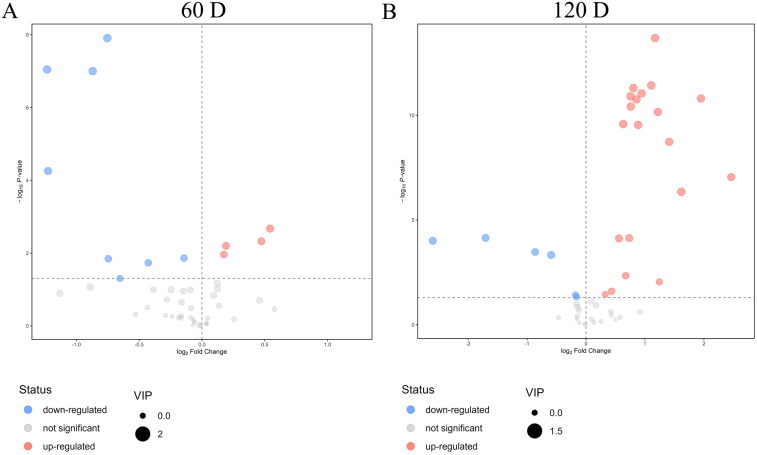
Volcano plots showing the changes in medium- and long-chain fatty acids profile in the egg yolk of aged laying hens (*n* = 8). (A and B) represent the results of Volcano plots between the Chinese herbal formula (CHF) group and the CON group at days 60 and 120 of the trial, respectively. CON, birds fed a basal diet; CHF, birds fed a basal diet supplemented with 1% CHF.

**Figure 4.**
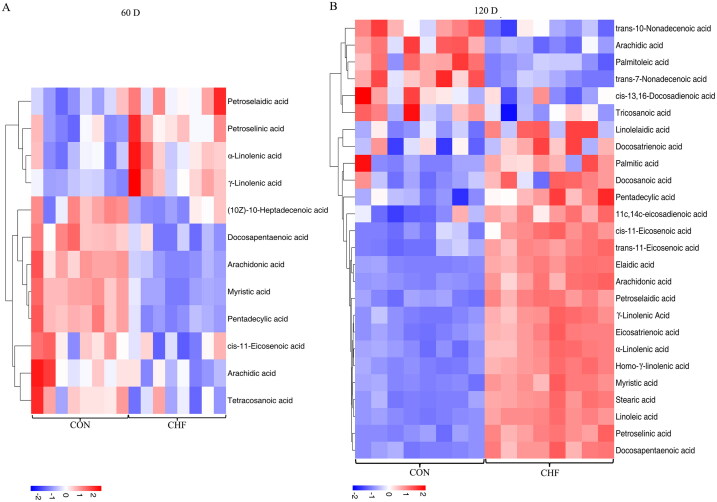
Analysis of differential fatty acids in egg yolks of aged laying hens (*n* = 8). Heatmap of differential medium- and long-chain fatty acids in egg yolks of aged laying hens at days 60 (A) and 120 (B) of the trial. CON, birds fed a basal diet; CHF, birds fed a basal diet supplemented with 1% Chinese herbal formula.

The volcano plot visualized the up-regulated and down-regulated metabolites in the CHF group compared with the CON group (Figure 3(A,B)). The heat map of differential metabolites (*p* < .05) is shown in Figure 4. At day 60 of the trial, 12 differential metabolites were identified in the CON and CHF groups, including petroselaidic acid (C18:1n-12t), petroselinic acid (C18:1n-12c), α-linolenic acid (C18:3n-3), γ-linolenic acid (C18:3n-6), (10Z)-10-heptadecenoic acid (C17:1n-7c), docosapentaenoic acid (C22:5n-3), arachidonic acid (C20:4n-6), myristic acid (C14:0), pentadecylic acid (C15:0), cis-11-eicosenoic acid (C20:1n-9c), arachidic acid (C20:0), and tetracosanoic acid (C24:0) (Figure 4(A)). At day 120 of the trial, 26 differential metabolites were identified in the CON and CHF groups, including trans-10-nonadecenoic acid (C19:1n-9t), C20:0, palmitoleic acid (C16:1n-7c), trans-7-nonadecenoic acid (C19:1n-12t), cis-13,16-docosadienoic acid (C22:2n-6), tricosanoic acid (C23:0), linolelaidic acid (C18:2n-6t), docosatrienoic acid (C22:3n-3), palmitic acid (C16:0), docosanoic acid (C22:0), C15:0, 11c,14c-eicosadienoic acid (C20:2n-6), C20:1n-9c, trans-11-eicosenoic acid (C20:1n-9t), elaidic acid (C18:1n-9t), C20:4n-6, C18:1n-12t, C18:3n-6, eicosatrienoic acid (C20:3n-3), C18:3n-3, homo-γ-linolenic acid (C20:3n-6), C14:0, stearic acid (C18:0), linoleic acid (C18:2n-6c), C18:1n-12c, and C22:5n-3 (Figure 4(B)).

To further identify the key differential metabolites, a two-way ANOVA was performed (Table 4). There were interactions (*p* < .05) between experimental time and CHF treatment for C7:0, C14:0, C15:0, C16:0, C18:0, C20:0, C16:1n-7c, C18:1n-12c, C18:1n-12t, C18:1n-9t, C19:1n-9t, C19:1n-12t, C20:1n-9c, C20:1n-9t, C18:2n-6t, C18:2n-6c, C18:3n-3, C18:3n-6, C20:2n-6, C20:3n-6, C20:3n-3, C20:4n-6, and C22:5n-3. Among these fatty acids, the concentrations of C14:0, C15:0, C16:0, C18:0, C18:1n-12c, C18:1n-12t, C18:1n-9t, C20:1n-9c, C20:1n-9t, C18:2n-6t, C18:2n-6c, C18:3n-3, C18:3n-6, C20:2n-6, C20:3n-6, C20:3n-3, C20:4n-6, and C22:5n-3 were increased in the D120-CHF group than the D60-CON, D60-CHF, and D120-CON groups. The concentrations of C20:0, C16:1n-7c, C19:1n-9t, and C19:1n-12t were increased in the D120-CON group than the D60-CON, D60-CHF, and D120-CHF groups.

**Table 4.** Effects of dietary supplementation with Chinese herbal formula (CHF) on medium- and long-chain fatty acid composition in egg yolks of aged laying hens.

Items (ng/mg)	Day 60 of the trial		Day 120 of the trial		SEM	*p*-values		
	CON	CHF	CON	CHF		Time	Diet	Time × Diet
SFA								
Hexanoic acid, C6:0	1.33	1.13	1.34	1.24	0.123	.829	.560	.846
Heptanoic acid, C7:0	0.30	0.14	0.19	0.26	0.028	.904	.369	.044
Undecanoic acid, C11:0	0.48	0.39	0.32	0.61	0.067	.801	.460	.173
Lauric acid, C12:0	0.35	0.24	7.12	8.49	1.038	<.001	.704	.658
Tridecylic acid, C13:0	1.44	1.07	1.67	1.83	0.159	.130	.737	.406
Myristic acid, C14:0	1.41^b^	0.84^d^	1.17^c^	2.11^a^	0.086	<.001	<.001	<.001
Pentadecylic acid, C15:0	0.53^a^	0.23^c^	0.36^b^	0.60^a^	0.029	.001	.230	<.001
Palmitic acid, C16:0	31.37^bc^	25.85^c^	44.31^b^	59.90^a^	2.931	<.001	.181	.008
Heptadecanoic acid, C17:0	0.13	0.12	0.16	0.14	0.003	<.001	.027	.438
Stearic acid, C18:0	17.64^c^	19.23^c^	24.09^b^	40.66^a^	1.661	<.001	<.001	<.001
Arachidic acid, C20:0	0.90^b^	0.54^b^	3.37^a^	1.04^b^	0.227	<.001	<.001	<.001
Heneicosanoic acid, C21:0	12.94	13.27	19.40	17.50	0.791	<.001	.549	.396
Docosanoic acid, C22:0	2.18	2.07	2.60	6.16	0.546	.026	.082	.065
Tricosanoic acid, C23:0	8.85	6.77	14.32	12.64	0.631	<.001	.012	.775
Tetracosanoic acid, C24:0	14.68	10.92	20.99	18.84	1.090	<.001	.106	.651
MUFA								
10(Z)-Pentadecenoic acid, C15:1n-5c	0.22	0.22	0.12	0.13	0.013	<.001	.729	.958
(10E)-10-Pentadecenoic acid, C15:1n-5t	0.29	0.39	0.26	0.19	0.032	.063	.771	.158
Palmitoleic acid, C16:1n-7c	0.64^c^	0.57^c^	2.90^a^	1.92^b^	0.182	<.001	<.001	<.001
(10Z)-10-Heptadecenoic acid, C17:1n-7c	0.57	0.51	1.35	1.22	0.070	<.001	.041	.390
trans-10-Heptadecenoic acid, C17:1n-7t	0.45	0.38	1.05	0.97	0.056	<.001	.042	.879
Petroselinic acid, C18:1n-12c	0.09^c^	0.10^b^	0.09^c^	0.15^a^	0.005	<.001	<.001	<.001
Oleic acid, C18:1n-9c	0.12	0.12	0.22	0.21	0.012	<.001	.953	.988
Petroselaidic acid, C18:1n-12t	0.22^c^	0.31^b^	0.22^c^	0.86^a^	0.049	<.001	<.001	<.001
Elaidic acid, C18:1n-9t	3.32^c^	3.54^c^	5.05^b^	10.89^a^	0.556	<.001	<.001	<.001
trans-10-Nonadecenoic acid, C19:1n-9t	0.45^b^	0.43^b^	1.22^a^	0.67^b^	0.065	<.001	<.001	<.001
trans-7-Nonadecenoic acid, C19:1n-12t	0.27^b^	0.27^b^	1.34^a^	0.22^b^	0.097	<.001	<.001	<.001
cis-11-Eicosenoic acid, C20:1n-9c	0.12^b^	0.08^b^	0.09^b^	0.49^a^	0.032	<.001	<.001	<.001
trans-11-Eicosenoic acid, C20:1n-9t	0.20^b^	0.20^b^	0.24^b^	0.64^a^	0.035	<.001	<.001	<.001
Erucic acid, C22:1n-9c	0.33	0.32	0.35	0.34	0.019	.517	.791	.934
Brassidic acid, C22:1n-9t	0.14	0.08	0.12	0.17	0.014	.185	.700	.060
Nervonic acid, C24:1n-9c	7.18	7.40	9.88	10.45	0.289	<.001	.141	.509
PUFA								
Linolelaidic acid, C18:2n-6t	0.06^c^	0.05^c^	0.10^b^	0.16^a^	0.009	<.001	.012	.003
Linoleic acid, C18:2n-6c	15.87^c^	17.27^c^	23.41^b^	52.72^a^	2.698	<.001	<.001	<.001
α-Linolenic acid, C18:3n-3	0.49^c^	0.55^c^	0.90^b^	1.40^a^	0.065	<.001	<.001	<.001
γ-Linolenic acid, C18:3n-6	0.16^d^	0.23^c^	0.56^b^	1.04^a^	0.063	<.001	<.001	<.001
11c,14c-Eicosadienoic acid, C20:2n-6	0.23^c^	0.22^c^	0.53^b^	0.79^a^	0.044	<.001	<.001	<.001
Homo-γ-linolenic acid, C20:3n-6	0.35^c^	0.31^c^	0.60^b^	1.15^a^	0.061	<.001	<.001	<.001
Eicosatrienoic acid, C20:3n-3	0.39^c^	0.35^c^	0.62^b^	1.05^a^	0.051	<.001	<.001	<.001
Arachidonic acid, C20:4n-6	10.29^b^	5.63^c^	10.58^b^	32.46^a^	1.903	<.001	<.001	<.001
5Z,8Z,11Z,14Z,17Z-Eicosapentaenoic acid, C20:5n-3	0.40	0.44	0.44	0.50	0.013	.037	.060	.738
cis-13,16-Docosadienoic acid, C22:2n-6	0.12	0.11	0.13	0.11	0.002	.431	.034	.494
Docosatrienoic acid, C22:3n-3	8.83	13.18	16.63	20.87	1.371	.003	.079	.981
7,10,13,16-Docosatetraenoic acid, C22:4n-6	2.04	2.43	1.66	2.35	0.350	.751	.463	.842
Osbond acid, C22:5n-6	0.35	0.30	0.25	0.37	0.041	.846	.647	.346
Docosapentaenoic acid, C22:5n-3	1.06^c^	0.45^d^	1.87^b^	4.35^a^	0.270	<.001	<.001	<.001

Data are presented as means with their SEM (*n* = 8).

^a−d^ Different letters indicate significant differences among the four groups (*p* < .05). CON: birds fed a basal diet; CHF: birds fed a basal diet supplemented with 1% CHF; SFA: saturated fatty acids; MUFA: monounsaturated fatty acids; PUFA: polyunsaturated fatty acids.

The concentrations of lauric acid (C12:0), heptadecanoic acid (C17:0), heneicosanoic acid (C21:0), docosanoic acid (C22:0), C23:0, C24:0, C17:1n-7c, oleic acid (C18:1n-9c), nervonic acid (C24:1n-9c), 5Z,8Z,11Z,14Z,17Z-eicosapentaenoic acid (C20:5n-3), and C22:3n-3 were increased with the age of laying hens (Table 4). However, the concentration of 10(Z)-pentadecenoic acid (C15:1n-5c) was decreased with the age of laying hens. In addition, dietary CHF supplementation decreased the concentrations of C17:0, C23:0, C17:1n-7c, trans-10-heptadecenoic acid (C17:1n-7t), and C22:2n-6.

**Figure 5.**
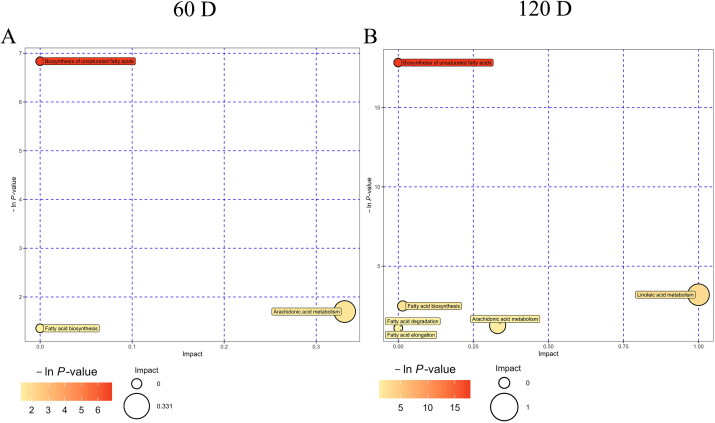
The scatter plots of the impact and enrichment of the metabolic pathway for all the matching significant medium- and long-chain fatty acids in egg yolks of aged laying hens between the Chinese herbal formula (CHF) group and the CON group at days 60 (A) and 120 (B) of the trial (*n* = 8). Metabolic pathway enrichment bubble plot: the vertical coordinate with the bubble indicates the *P*-value of the enrichment analysis, taking the negative logarithm of the natural number e as the base (i.e. for the –ln *p*-value, darker colors indicate a smaller *p*-value and a more significant enrichment). The larger the bubble size, the larger the influence factor; the darker the color, the smaller the *p*-value, and the more significant the enrichment degree.

The KEGG database was used for metabolic pathway enrichment analysis of differential metabolites to explore which metabolic pathways were affected by dietary CHF supplementation (Figure 5). At day 60 of the trial, dietary CHF supplementation enriched three differential metabolic pathways, including biosynthesis of unsaturated fatty acids, arachidonic acid metabolism, and fatty acid biosynthesis compared with the CON group. At day 120 of the trial, dietary CHF supplementation enriched six differential metabolic pathways, including biosynthesis of unsaturated fatty acids, linoleic acid metabolism, fatty acid biosynthesis, arachidonic acid metabolism, fatty acid degradation, and fatty acid elongation compared with the CON group.

To evaluate the nutritional value of the fatty acids profile in egg yolks, we classified the various fatty acids and calculated the health-related indicators (Table 5). The SFA, MUFA, PUFA, and PUFA/SFA levels were increased (*p* < .05) with the increased age of laying hens. Dietary CHF supplementation increased (*p* < .05) the MUFA, PUFA, and PUFA/SFA levels compared with the CON group. The values of SFA, MUFA, PUFA, and PUFA/SFA in the D120-CHF group were higher (*p* < .05) than those in the D60-CON, D60-CHF, and D120-CON groups. Moreover, the n-3 and n-6 PUFA concentrations were increased (*p* < .05) with the age of laying hens and CHF treatment. The n-6 PUFA concentration was higher (*p* < .05) in the D120-CHF group than that in the other three groups, as well as the n-6/n-3 PUFA concentration than that in the D120-CON group.

**Table 5.** Effects of dietary supplementation with Chinese herbal formula (CHF) on medium- and long-chain fatty acid indexes in egg yolks of aged laying hens.

Items (ng/mg)	Day 60 of the trial		Day 120 of the trial		SEM	*p*-values		
	CON	CHF	CON	CHF		Time	Diet	Time × Diet
SFA	94.53^c^	82.79^c^	141.41^b^	172.01^a^	7.078	<.001	.134	.002
MUFA	14.61^c^	14.92^c^	24.50^b^	29.52^a^	1.174	<.001	<.001	<.001
PUFA	40.63^c^	41.54^c^	58.29^b^	119.30^a^	5.945	<.001	<.001	<.001
PUFA/SFA	0.44^b^	0.50^b^	0.42^b^	0.70^a^	0.025	.006	<.001	.002
n-3 PUFA	11.17	14.98	20.47	28.16	1.603	<.001	.021	.417
n-6 PUFA	29.46^c^	26.56^c^	37.82^b^	91.14^a^	4.763	<.001	<.001	<.001
n-6 /n-3 PUFA	2.79^ab^	2.39^ab^	1.91^b^	3.28^a^	0.151	.988	.070	.002
AI	0.68	0.54	0.68	0.52	0.028	.814	.005	.873
TI	0.93	0.81	0.76	0.71	0.039	.085	.268	.581
HH	0.95^b^	0.92^b^	0.87^b^	1.51^a^	0.063	.008	.002	.001
HPI	1.59	1.91	1.52	2.01	0.078	.932	.010	.574
DFA	72.87^c^	75.69^c^	106.88^b^	189.47^a^	8.645	<.001	<.001	<.001

Data are presented as means with their SEM (*n* = 8).

^a−c^ Different letters indicate significant differences among the four groups (*p* < .05). CON: birds fed a basal diet; CHF: birds fed a basal diet supplemented with 1% Chinese herb formula; SFA: saturated fatty acids; MUFA: monounsaturated fatty acids; PUFA: polyunsaturated fatty acids; AI: atherogenicity index; TI: thrombogenic index; HH: hypocholesterolaemic to hypercholesterolaemic ratio; HPI: health promotion index; DFA: desirable fatty acids.

Regarding healthy indexes (Table 5), there was an increase (*p* < .05) in HH ratio and DFA at day 120 of the trial. Dietary CHF supplementation increased the HH ratio, HPI, and DFA while decreasing AI level when compared with those fed with a basal diet (*p* < .05). There was an interaction (*p* < .05) between experimental time and CHF treatment for the HH ratio and DFA concentration. The HH ratio and DFA concentration in the D120-CHF group were higher (*p* < .05) than those in the other three groups.

## Discussion

Numerous metabolic disorders occur during the late laying period, which enormously affects the body health and egg quality of laying hens, resulting in economic losses. Several factors are related to fatty acid in yolks, such as the age of laying hens and diet composition (Koppenol et al., 2014). Different nutritional strategies have been used to improve the body health and egg quality of laying hens during the late laying period over the previous few decades. Therefore, the present study evaluated the effects of dietary CHF supplementation on the plasma biochemical parameters and yolk fatty acids profiles of Xinyang-black feather laying hens at 54 and 62 weeks of age (in the late laying period), and the results showed that dietary CHF could significantly improve the nutritional value of the egg yolk.

The egg yolk contains more than 60% lipids (including fatty acids) of its dry weight, which are the main components of egg yolk (Golzar Adabi et al. 2013). The composition and concentrations of fatty acids in egg yolks affect the physical and chemical properties and flavor of eggs and are important indicators of the nutritional value of eggs (Nielsen 1998). In the present study, dietary CHF supplementation increased the total PUFA and n-3 PUFA (including α-linolenic acid and 5Z,8Z,11Z,14Z,17Z-eicosapentaenoic acid) in egg yolks compared with the CON group at days 60 and 120 of the trial. Meanwhile, the concentrations of n-6 PUFAs such as linoleic acid, γ-linolenic acid, docosatrienoic acid, and arachidonic acid in egg yolks in the CHF group were higher compared with the CON group at day 120 of the trial, especially arachidonic acid. The γ-linolenic acid can be converted into docosahexanoate, known as ‘brain gold’, improving brain health, reducing blood lipids, and delaying aging (Horrocks and Yeo 1999). Linoleic acid can be converted to γ-linolenic acid in the human body, which in turn becomes arachidonic acid. In addition, linoleic acid also reduces the serum levels of cholesterol and low-density lipoprotein cholesterol, which could affect lipoprotein metabolism and reduce the incidence of cardiovascular disease (Marangoni et al. 2020). Arachidonic acid can produce eicosanoidin, a bioactive inflammatory substance that produces inflammatory mediators, thus regulating the inflammatory response (Burns et al. 2018). A previous study showed that the top 20 pathways of KEGG functional enrichment analysis of TM included arachidonic acid metabolism (Liu et al. 2022). Consistent with these results, our pathway enrichment analysis in metabolomics confirmed the effects of dietary CHF supplementation on the biosynthesis of unsaturated fatty acids (UFA) and arachidonic acid metabolism. Therefore, the TM may partly contribute to the promising potential of CHF for the regulation of PUFA in egg yolks.

MUFAs are the main components of TG, cholesterol esters (cellular membrane components and precursors of steroid hormones and bile acids), wax esters, and phospholipids comprising cellular membranes (Ravaut et al. 2020). In the present study, dietary CHF supplementation significantly increased the total amount of MUFA in a time-dependent manner and several MUFA concentrations, including elaidic acid, petroselinic acid, and petroselaidic acid. Moreover, dietary CHF supplementation significantly decreased SFAs concentrations, including myristic acid, pentadecylic acid, and palmitic acid in egg yolks at day 60 of the trial, whereas increased the concentrations of these SFAs in egg yolks at day 120 of the trial. The SFAs are considered a predisposing factor for cardiovascular disease, associated with higher cholesterol, hyperlipidemia, and obesity (Virtanen et al. 2016). Nevertheless, other studies have reported that SFAs play a significant role in the growth and metabolism of cells (Rioux and Legrand 2007). However, the inconsistencies of these results on SFAs concentrations in the present study might be attributed to the treatment time and the age of the laying hens.

The source, synthesis, and transportation of fatty acids in egg yolks are closely related to the liver and ovary. Polysaccharides, alkaloids, flavonoids, UFA, and other bioactive components in Chinese herbs can play crucial roles in lipid-lowering and related regulating metabolism. Ajugol, the LJ extracts, ameliorates hepatic steatosis *via* mTOR-TFEB-mediated lysosomal biogenesis (Zhang et al. 2021). Oleanolic acid in LL has hypoglycemic, hypolipidemic, and antioxidant effects on diabetic rats and protects liver function by inhibiting alloxan-induced damage (Gao et al. 2009). The TM extracts have been found useful for hypolipidemic effects due to their down-regulated expression of fatty acid synthase and the inhibiting activity of acetyl-coenzyme A carboxylase through the phosphorylation of AMP-activated protein kinase (Liu et al. 2014). *In vivo* and *in vitro* studies showed that SM extracts, such as tanshinone IIA and salvianolate, induce anti-oxidative and anti-inflammatory effects (Ren et al. 2019). The regulatory effects of dietary CHF on fatty acids in egg yolks may be attributed to the characteristics of Chinese herb ingredients as mentioned earlier not given by differences in the composition of the diets actually provided (Table 2). These bioactive ingredients in the CHF might be involved in regulating the liver and ovarian performance of laying hens, which warrants further in-depth study.

The nutritional values of egg yolk are attributed to its fat quality and fatty acid composition, and a balanced intake of fatty acids is essential for reducing the risk of atherosclerosis, cardiovascular disease, and other related diseases (Shahidi and Ambigaipalan 2018). The n-6 and n-3 PUFA and n-6/n-3 PUFA are the crucial fatty acids that control the hypocholesterolemic index. The n-3 PUFA plays a major role in regulating the thrombogenic index, whereas the n-6 PUFA is dominant for the atherogenic. Moreover, the n-6/n-3 PUFA ratio < 4.0 can prevent cardiovascular diseases (Wołoszyn et al. 2020), as also indicated in the present study. The European Health Agency recommended ratio of PUFA/SFA is higher than 0.45 in a daily diet (Scollan et al. 2006). The PUFA/SFA ratio in the CHF groups was higher than 0.45, even up to 0.70 at day 120 of the trial, whereas it was lower than 0.45 in the CON group. These results suggest that dietary CHF supplementation improved the egg fatty acids profiles.

The AI and TI indexes reflect trends in platelet aggregation (Chen and Liu 2020), which commonly recommended for human consumption are less than 0.5 and 1.0, respectively, in food products (Wołoszyn et al. 2020). In the present study, dietary CHF supplementation decreased the AI index in egg yolks from 0.68 to 0.52. Also, the TI index ranged from 0.71 to 0.81 in the CHF groups, which was within the recommended range. The HH ratio is related to cholesterol metabolism; generally, a higher HH value is associated with better health (Dernekbaşı and Karayücel 2021). Moreover, HPI and DFA indexes are beneficial for human health (Medeiros et al. 2014; Hanuš et al. 2018). In the present study, dietary CHF supplementation significantly increased the HH ratio, HPI, and DFA in egg yolks. These results indicate that dietary CHF supplementation could enhance the nutritional values of egg yolks. Leonurin isolated from LJ and tanshinone I, tanshinone IIA, cryptotanshinone, salvianolic acid B, ferulic acid, and salvianolic acid A isolated from SM, showed protective effects on the cardiovascular disease (Wojtyniak et al. 2013; Zhang et al. 2022). These components in the CHF may play a potential role in fatty acid changes in egg yolks.

## Conclusion

In summary, dietary CHF supplementation could increase the concentrations of fatty acids and optimize the fatty acid composition in egg yolks without affecting the plasma biochemical parameters in aged laying hens. Dietary CHF supplementation increased MUFA, PUFA/SFA, n-6/n-3 PUFA, HH, and DFA while decreased AI and TI indexes in the egg yolk. These findings will provide the guiding significance of using CHF as feed additives in poultry production to improve the nutritional value of eggs.

## References

[CIT0001] Abbood AA, Kassim AB, Jawad HSA, Manap YA, Sazili AQ. 2017. Effects of feeding the herb *Borreria latifolia* on the meat quality of village chickens in Malaysia. Poult Sci. 96(6):1767–1782. doi: 10.3382/ps/pew460. 28204764

[CIT0002] Batkowska J, Drabik K, Brodacki A, Czech A, Adamczuk A. 2021. Fatty acids profile, cholesterol level and quality of table eggs from hens fed with the addition of linseed and soybean oil. Food Chem. 334:127612. doi: 10.1016/j.foodchem.2020.127612. 32731121

[CIT0003] Burns JL, Nakamura MT, Ma DWL. 2018. Differentiating the biological effects of linoleic acid from arachidonic acid in health and disease. Prostaglandins Leukot Essent Fatty Acids. 135:1–4. doi: 10.1016/j.plefa.2018.05.004. 30103919

[CIT0004] Chen G, Li Z, Liu S, Tang T, Chen Q, Yan Z, Peng J, Yang Z, Zhang G, Liu Y, et al. 2023. Fermented Chinese herbal medicine promoted growth performance, intestinal health, and regulated bacterial microbiota of weaned piglets. Animals. 13(3):476. doi: 10.3390/ani13030476. 36766365 PMC9913397

[CIT0005] Chen J, Liu H. 2020. Nutritional indices for assessing fatty acids: a mini-review. Int J Mol Sci. 21(16):5695. doi: 10.3390/ijms21165695. 32784511 PMC7460856

[CIT0006] Dai D, Qi GH, Wang J, Zhang HJ, Qiu K, Wu SG. 2022. Intestinal microbiota of layer hens and its association with egg quality and safety. Poult Sci. 101(9):102008. doi: 10.1016/j.psj.2022.102008. 35841638 PMC9289868

[CIT0007] Dernekbaşı S, Karayücel İ. 2021. Effect of alternate feeding with fish oil- and peanut oil-based diets on the growth and fatty acid compositions of European seabass fingerlings (*Dicentrarchus labrax*) in the recirculated systems. Aquac Res. 52(7):3137–3147. doi: 10.1111/are.15160.

[CIT0008] Gao D, Li Q, Li Y, Liu Z, Fan Y, Liu Z, Zhao H, Li J, Han Z. 2009. Antidiabetic and antioxidant effects of oleanolic acid from *Ligustrum lucidum* Ait in alloxan-induced diabetic rats. Phytother Res. 23(9):1257–1262. doi: 10.1002/ptr.2603. 19274687

[CIT0009] Golzar Adabi SH, Ahbab M, Fani AR, Hajbabaei A, Ceylan N, Cooper RG. 2013. Egg yolk fatty acid profile of avian species-influence on human nutrition. J Anim Physiol Anim Nutr. 97(1):27–38. doi: 10.1111/j.1439-0396.2011.01239.x. 22035443

[CIT0010] Gu YF, Chen YP, Jin R, Wang C, Wen C, Zhou YM. 2021. A comparison of intestinal integrity, digestive function, and egg quality in laying hens with different ages. Poult Sci. 100(3):100949. doi: 10.1016/j.psj.2020.12.046. 33652523 PMC7936206

[CIT0011] Han K, Lan W, Hu X, Cui Y, Kong X. 2023. Effects of compound Chinese herb ultrafine powder on antioxidant ability and related gene expression in laying hens. Acta Vet Et Zootech Sinica. 9:3784–3792. doi: 10.11843/j.issn.0366-6964.2023.09.018.

[CIT0012] Han K, Lin W, Meng C, Lan W, Hu X, Cui Y, Kong X. 2023. Effects of compound Chinese medicine ultrafine powder on egg production, reproductive hormones, and related gene expression in laying hens. J South China Agri Univ. 3:374–381. doi: 10.7671/j.issn.1001-411X.202206047.

[CIT0013] Hanuš O, Samková E, Křížová L, Hasoňová L, Kala R. 2018. Role of fatty acids in milk fat and the influence of selected factors on their variability-A review. Molecules. 23(7):1636. doi: 10.3390/molecules23071636. 29973572 PMC6100482

[CIT0014] Horrocks LA, Yeo YK. 1999. Health benefits of docosahexaenoic acid (DHA). Pharmacol Res. 40(3):211–225. doi: 10.1006/phrs.1999.0495. 10479465

[CIT0015] Hou Y, Jiang JG. 2013. Origin and concept of medicine food homology and its application in modern functional foods. Food Funct. 4(12):1727–1741. doi: 10.1039/c3fo60295h. 24100549

[CIT0016] Koppenol A, Delezie E, Aerts J, Willems E, Wang Y, Franssens L, Everaert N, Buyse J. 2014. Effect of the ratio of dietary n-3 fatty acids eicosapentaenoic acid and docosahexaenoic acid on broiler breeder performance, egg quality, and yolk fatty acid composition at different breeder ages. Poult Sci. 93(3):564–573. doi: 10.3382/ps.2013-03320. 24604849

[CIT0017] Kovacs-Nolan J, Phillips M, Mine Y. 2005. Advances in the value of eggs and egg components for human health. J Agric Food Chem. 53(22):8421–8431. doi: 10.1021/jf050964f. 16248532

[CIT0018] Liu C, Yang T, Zhao Z, Liu TC, Li K, Liu J, Zhou P. 2022. Effects of particle size reduction combined with β-cyclodextrin on the *in vitro* dissolution and *in vivo* relative bioavailability of ginsenosides in *Panax ginseng*. Food Funct. 13(21):10882–10894. doi: 10.1039/d2fo01098d. 36222359

[CIT0019] Liu FJ, Yang J, Chen XY, Yu T, Ni H, Feng L, Li P, Li HJ. 2022. Chemometrics integrated with *in silico* pharmacology to reveal antioxidative and anti-inflammatory markers of dandelion for its quality control. Chin Med. 17(1):125. doi: 10.1186/s13020-022-00679-4. 36333721 PMC9636813

[CIT0020] Liu XT, Lin X, Mi YL, Zeng WD, Zhang CQ. 2018. Age-related changes of yolk precursor formation in the liver of laying hens. J Zhejiang Univ Sci B. 19(5):390–399. doi: 10.1631/jzus.B1700054. 29732750 PMC5962516

[CIT0021] Liu Y, Azad MAK, Zhao X, Zhu Q, Kong X. 2022. Dietary crude protein levels alter diarrhea incidence, immunity, and intestinal barrier function of Huanjiang mini-pigs during different growth stages. Front Immunol. 13:908753. doi: 10.3389/fimmu.2022.908753. 35874746 PMC9301461

[CIT0022] Liu YJ, Shieh PC, Lee JC, Chen FA, Lee CH, Kuo SC, Ho CT, Kuo DH, Huang LJ, Way TD. 2014. Hypolipidemic activity of *Taraxacum mongolicum* associated with the activation of AMP-activated protein kinase in human HepG2 cells. Food Funct. 5(8):1755–1762. doi: 10.1039/c4fo00183d. 24903219

[CIT0023] Lv W, Liu C, Zeng Y, Li Y, Chen W, Shi D, Guo S. 2019. Explore the potential effect of natural herbals to resist Newcastle disease virus. Poult Sci. 98(5):1993–1999. doi: 10.3382/ps/pey557. 30566670

[CIT0024] Marangoni F, Agostoni C, Borghi C, Catapano AL, Cena H, Ghiselli A, La Vecchia C, Lercker G, Manzato E, Pirillo A, et al. 2020. Dietary linoleic acid and human health: focus on cardiovascular and cardiometabolic effects. Atherosclerosis. 292:90–98. doi: 10.1016/j.atherosclerosis.2019.11.018. 31785494

[CIT0025] Medeiros E, Queiroga R, Oliveira M, Medeiros A, Sabedot M, Bomfim M, Madruga M. 2014. Fatty acid profile of cheese from dairy goats fed a diet enriched with castor, sesame and faveleira vegetable oils. Molecules. 19(1):992–1003. doi: 10.3390/molecules19010992. 24434672 PMC6270699

[CIT0026] Nielsen H. 1998. Hen age and fatty acid composition of egg yolk lipid. Br Poult Sci. 39(1):53–56. doi: 10.1080/00071669889394. 9568299

[CIT0027] Obianwuna UE, Oleforuh-Okoleh VU, Wang J, Zhang HJ, Qi GH, Qiu K, Wu SG. 2022. Natural products of plants and animal origin improve albumen quality of chicken eggs. Front Nutr. 9:875270. doi: 10.3389/fnut.2022.875270. 35757269 PMC9226613

[CIT0028] Omri B, Chalghoumi R, Izzo L, Ritieni A, Lucarini M, Durazzo A, Abdouli H, Santini A. 2019. Effect of dietary incorporation of linseed alone or together with tomato-red pepper mix on laying hens’ egg yolk fatty acids profile and health lipid indexes. Nutrients. 11(4):813. doi: 10.3390/nu11040813. 30974860 PMC6521111

[CIT0029] Ravaut G, Légiot A, Bergeron K-F, Mounier C. 2020. Monounsaturated fatty acids in obesity-related inflammation. Int J Mol Sci. 22(1):330. doi: 10.3390/ijms22010330. 33396940 PMC7795523

[CIT0030] Ren J, Fu L, Nile SH, Zhang J, Kai G. 2019. *Salvia miltiorrhiza* in treating cardiovascular diseases: A review on its pharmacological and clinical applications. Front Pharmacol. 10:753. doi: 10.3389/fphar.2019.00753. 31338034 PMC6626924

[CIT0031] Rioux V, Legrand P. 2007. Saturated fatty acids: Simple molecular structures with complex cellular functions. Curr Opin Clin Nutr Metab Care. 10(6):752–758. doi: 10.1097/MCO.0b013e3282f01a75. 18089958

[CIT0032] Scollan N, Hocquette J-F, Nuernberg K, Dannenberger D, Richardson I, Moloney A. 2006. Innovations in beef production systems that enhance the nutritional and health value of beef lipids and their relationship with meat quality. Meat Sci. 74(1):17–33. doi: 10.1016/j.meatsci.2006.05.002. 22062713

[CIT0033] Shahidi F, Ambigaipalan P. 2018. Omega-3 polyunsaturated fatty acids and their health benefits. Annu Rev Food Sci Technol. 9(1):345–381. doi: 10.1146/annurev-food-111317-095850. 29350557

[CIT0034] Virtanen JK, Mursu J, Virtanen HEK, Fogelholm M, Salonen JT, Koskinen TT, Voutilainen S, Tuomainen TP. 2016. Associations of egg and cholesterol intakes with carotid intima-media thickness and risk of incident coronary artery disease according to apolipoprotein E phenotype in men: The Kuopio ischaemic heart disease risk factor study. Am J Clin Nutr. 103(3):895–901. doi: 10.3945/ajcn.115.122317. 26864369

[CIT0035] Wang Y, Yang H, Chen L, Jafari M, Tang J. 2021. Network-based modeling of herb combinations in traditional Chinese medicine. Brief Bioinform. 22:1–13. doi: 10.1093/bib/bbab106 33834186 PMC8425426

[CIT0036] Wojtyniak K, Szymański M, Matławska I. 2013. *Leonurus cardiaca* L. (motherwort): A review of its phytochemistry and pharmacology. Phytother Res. 27(8):1115–1120. doi: 10.1002/ptr.4850. 23042598

[CIT0037] Wołoszyn J, Haraf G, Okruszek A, Wereńska M, Goluch Z, Teleszko M. 2020. Fatty acid profiles and health lipid indices in the breast muscles of local polish goose varieties. Poult Sci. 99(2):1216–1224. doi: 10.1016/j.psj.2019.10.026. 32036970 PMC7587679

[CIT0038] Zhang DY, Peng RQ, Wang X, Zuo HL, Lyu LY, Yang FQ, Hu YJ. 2022. A network pharmacology-based study on the quality control markers of antithrombotic herbs: using *Salvia miltiorrhiza*-*Ligusticum chuanxiong* as an example. J Ethnopharmacol. 292:115197. doi: 10.1016/j.jep.2022.115197. 35331879

[CIT0039] Zhang H, Lu J, Liu H, Guan L, Xu S, Wang Z, Qiu Y, Liu H, Peng L, Men X. 2021. Ajugol enhances TFEB-mediated lysosome biogenesis and lipophagy to alleviate non-alcoholic fatty liver disease. Pharmacol Res. 174:105964. doi: 10.1016/j.phrs.2021.105964. 34732369

[CIT0040] Zhang J, Yu H, Zhang H, Zhao Q, Si W, Qin Y, Zhang J. 2023. Dietary epimedium extract supplementation improves intestinal functions and alters gut microbiota in broilers. J Anim Sci Biotechnol. 14(1):14. doi: 10.1186/s40104-022-00812-1. 36653873 PMC9847172

[CIT0041] Zhu Q, Song M, Azad MAK, Ma C, Yin Y, Kong X. 2022. Probiotics and synbiotics addition to Bama mini-pigs’ diet improve carcass traits and meat quality by altering plasma metabolites and related gene expression of offspring. Front Vet Sci. 9:779745. doi: 10.3389/fvets.2022.779745. 35873696 PMC9301501

